# Evaluating digital nudge interventions for the promotion of cancer screening behavior: a systematic review and meta-analysis

**DOI:** 10.1186/s12916-025-04028-8

**Published:** 2025-04-14

**Authors:** Fangfang Wang, Yonglin Li, Chenxing Zhang, Rachel Arbing, Wei-Ti Chen, Feifei Huang

**Affiliations:** 1 School of Nursing, Fujian Medical University, No 1, Xueyu Road, Minhou County, Fuzhou, Fujian 350108 China; 2 School of Nursing, University of California los Angeles, 700 Tiverton Avenue, Los Angeles, CA 90095 USA

**Keywords:** Digital nudge, Cancer screening behavior, Meta-analysis, Systematic review

## Abstract

**Background:**

Public adherence to cancer screening remains low and is influenced by both rational and non-rational factors, including decision biases that underestimate screening benefits. Digital nudge interventions have shown promise in promoting screening behaviors among at-risk populations, but systematic evidence is still lacking. This study aims to synthesize the effects of digital nudge interventions on promoting cancer screening behaviors in high-risk individuals.

**Methods:**

A systematic search of 10 electronic databases was conducted, and studies published before April 1, 2024, were included. Eligible studies were randomized controlled trials (RCTs) that compared the effects of digital nudge interventions on cancer screening behavior with those of a control group and reported at least one outcome. The risk of bias was evaluated using the Cochrane Risk of Bias tool. Data on cancer screening uptake rates were pooled using a random-effects model. Subgroup analyses were performed for cancer types, intervention media, delivery conditions, and sensitivity. The study identified digital nudge strategies via the MINDSPACE framework and explored their influence on screening behavior through the HSM.

**Results:**

Of the 14 randomized controlled trials included, 10 reported statistically significant results. The types of interventions in these studies were heterogeneous and available across multiple delivery channels based on the web, computer programmes, DVDs, telephones, patient navigation, or apps that tailored or served interactive information to participants to better understand screening risks and options. A random-effects model showed that digital nudge intervention strategies significantly improved adherence to cancer screening behavior (OR = 1.81, 95% CI = 1.35–2.44, *p* < 0.001). Differences between cancer types, intervention media, and delivery conditions were noted. Based on the MINDSPACE framework and HSM, eight nudge strategies were designed to promote screening behaviors, with the most common being the default strategy (*n* = 9). Most nudge tools were designed to leverage unconscious System 1 thinking, aiming to influence behavior in a more spontaneous and subtle way.

**Conclusions:**

While digital nudge interventions have demonstrated significant positive effects in promoting early cancer screening participation among high-risk individuals, their impact varies. More robust research is needed to address methodological limitations and facilitate broader adoption and application of these interventions.

**Supplementary Information:**

The online version contains supplementary material available at 10.1186/s12916-025-04028-8.

## Background

Cancer has emerged as the leading cause of death globally, affecting nearly 20 million people and claiming approximately 9.7 million lives in 2022 [1]. China also faces this alarming trend, with approximately 4,824,700 new cancer cases and 2,574,200 new cancer deaths reported in 2022 [2]. Despite remarkable progress in medical technology, cancer screening remains a crucial diagnostic tool, demonstrating the potential to prevent more than 40% of cancer-related deaths [3].

In response, many countries have proposed cancer screening guidelines and programs to enhance the uptake of screening among individuals at high risk, aiming to improve adherence to cancer screening. For instance, in 2002, the “Large National Lung Screening Trial” (NLST), supported by the National Cancer Institute in the United States, involved 53,456 high-risk participants who were offered one baseline screening and two annual screenings with low-dose computed tomography (LDCT) or chest radiography [4]. Similarly, the “Cancer Screening Project in Urban Areas of China”, initiated in 2012, aimed to provide 700,000 cost-free screenings over five years for various cancers, including breast, colorectal, cervical, and lung cancers [5].

Despite these concerted efforts, public adherence to cancer screening, as per recommendations, remains suboptimal. Taking lung cancer screening (LCS) as an example, reported adherence varies from 12 to 91% [6]. This fluctuation can be attributed to differences in institutional practices related to LCS program implementation, the screened populations, and the applied definition of screening adherence. Additionally, some studies have revealed that fewer than half of eligible individuals invited to participate in centralized or population-based cancer screening programs manage to complete all scheduled screening tests [7].

Recent evidence-based reviews suggest that cancer screening adherence is significantly influenced by factors encompassing individual characteristics, social influences, and health system challenges [8, 9]. These factors include low health literacy, fear of screening, feelings of embarrassment, a sense of distrust, a lack of perceived risk of cancer, and issues related to time and finances [10, 11]. Moreover, studies have shown that individuals’ decision-making and behaviors are often shaped by biased emotional, mental, and cognitive processing, such as cognitive inertia (“no symptoms, no screening”), fatalism, and the belief that health checkups are useless, rather than being solely determined by rational thinking, which leads to a decision bias of underestimating the potential but uncertain benefits of cancer screening which affects screening behaviors in the future.

This phenomenon aligns with the dual mechanism of the Heuristic-Systematic Model (HSM), which posits two pathways for processing information and forming attitudes: “System 1” and “System 2”. System 1 is fast, spontaneous, emotionally driven, and relies on unconscious, efficient cognition. In contrast, System 2 is slower, more deliberate, and rational and is associated with deep thinking and decision-making [12]. The HSM demonstrates how these two modes influence attitudes and decision-making across different contexts, emphasizing the critical role of irrational, affective cognition in motivating screening behavior among high-risk individuals.

To address non-rational factors in decision-making, nudging has been used to influence individual behavior. A nudge is defined as a predictable way to alter people’s behavior without restricting choices or significantly changing economic incentives [13]. This means that individuals retain their power of choice, while behaviors and choices conducive to intended outcomes are automatically encouraged. To date, nudging has demonstrated significant advantages in improving decision-making related to behaviors such as organ donation, weight loss, healthy eating, smoking and alcohol control, and cancer screening [14]. In the field of cancer screening, scholars have employed various nudging tools, such as decision nudges, navigation nudges, and framing effect nudges [15], to enhance the screening behavior of high-risk populations for breast cancer, colorectal cancer, cervical cancer, etc., with promising initial results.

Non-mandatory nudge strategies are emerging as effective tools to increase participation in cancer screening. The MINDSPACE framework, a widely adopted nudge theory, accurately captures the imperfect rationality of individual behaviors. It identifies nine key drivers: Messenger (M), Incentives (I), Norms (N), Defaults (D), Salience (S), Priming (P), Affect (A), Commitments (C), and Ego (E) [16]. The MINDSPACE framework emphasizes the role of environmental factors, information presentation, and individual psychological characteristics in decision-making by integrating multidimensional cognitive and behavioral analysis tools. It promotes a positive cognitive-to-behavioral shift and facilitates the adoption and sustainability of health behaviors. The framework has already been successfully applied in various public health areas, including cancer screening, vaccination, dietary improvements, exercise promotion, and smoking cessation programs [17, 18].

In the internet era, the concept of digital nudging has gained traction. Rooted in human–computer interaction, digital nudging refers to the use of various user interface design elements to guide people’s judgments and decisions. Digital nudging interventions aim to overcome barriers to cancer screening participation through timely reminders and by making information more accessible, often using behavioral science principles to influence decision-making [19]. These types of interventions are more likely to be socially and professionally acceptable than mandates are and have the potential to enhance awareness, engagement, and adherence to recommended cancer screening practices.

Current studies have yielded inconsistent or inconclusive results. For example, in 2017, Bowen et al.’s study achieved significant results in helping women make more informed health choices to improve their breast health through a web-based nudge intervention [20]. However, other studies have attempted to use digital nudge strategies to improve mammogram adherence among African American women and reported no significant differences over controls [21]. Therefore, it is crucial to assess the value of digital nudge interventions in cancer screening through a rigorous and evidence-based methodology.

Previous systematic reviews on nudge strategies have explored diverse areas, including efforts to improve the health of older people with mild cognitive impairment [22], chronic disease self-management [23], and HIV and malaria testing [24], but the behavioral dimension of screening for individuals at high risk of cancer has not been the focus. To date, only Richardson-Parry et al. [25] have cataloged the role of digital interventions in increasing screening participation among underserved populations, finding that effective digital intervention strategies can help reduce health inequalities in cancer screening [25]. However, their study did not specifically address nudging. Systematic summaries of the effects of digital nudge interventions are even more scarce. Given this context, a systematic literature review of the effectiveness of digital nudge interventions in promoting cancer screening is particularly important and urgent.

Our review aimed to identify the characteristics of digital nudge interventions that target early detection behaviors in cancer screening and to assess the effectiveness of these interventions on the basis of the MINDSPACE framework and Chaiken’s heuristic-systematic model (HSM) of information processing. This review provides a scientific basis for the development and optimization of future intervention strategies.

## Methods

### Protocol and registration

This systematic review was registered with the International Prospective Register of Systematic Reviews (PROSPERO) (registration number: CRD42023449526). The Preferred Reporting Items for Systematic Reviews and Meta-Analyses (PRISMA) guidelines were used to conduct this systematic review [26].

### Search strategy

We conducted a comprehensive search for published studies across 10 electronic databases, covering the English-language databases PubMed, Web of Science, CINAHL, Cochrane Library, and PsycINFO. Additionally, we included Chinese databases, namely the Chinese National Knowledge Infrastructure (CNKI), WanFang Database, China Science and Technology Journal Database (VIP database), SinoMed, and Chinese Medical Journal Database. A full search strategy was used for each database and the complete search string can be found in Additional file 1: Table S1. We considered studies published before April 1, 2024. We also performed a backward search of the reference lists of all included studies. Eligibility criteria were defined according to the population, intervention, comparison, outcomes, and study design framework (PICOS) inclusion criteria as outlined in Table 1.

**Table 1:** Inclusion and Exclusion Criteria

	Inclusion Criteria	Exclusion Criteria
P (population)	Adults eligible for cancer screening as per established guidelines. Studies on breast, cervical, colorectal, prostate, or lung cancer were included as screening guidelines already exist for these cancers	Exclusions encompassed commentaries, grey literature, duplicate articles, conference abstracts, letters, reviews, protocols, and e ditorials
I (intervention)	Study examines digital nudge interventions for cancer screening	
C (comparison)	Any comparator was acceptable, including delayed interventions, non-intervention groups (care-as-usual), non-digital nudge interventions	
O (outcomes)	Primary outcome is change in cancer screening early detection behavior (e.g., willingness to participate in cancer screening, participation rates)	
S (study design)	Randomized clinical trials reported in English or Chinese,and studies with multifaceted interventions as long as at least one component involved a digital nudge strategy	

### Study selection and quality assessment

Following predefined eligibility criteria, two independent reviewers screened titles and abstracts and excluded irrelevant articles. Full-text assessments were conducted for potentially relevant articles, with an independent methodological quality assessment performed by two researchers. The Cochrane Collaboration tool was used to assess RCTs, whereas the Risk of Bias in Nonrandomized Studies-of Interventions (ROBINS-I) criteria evaluated before-and-after studies [27]. The risk of bias for the included studies was categorized as low, medium, or high. Additionally, the certainty of evidence for outcomes was assessed using the GRADE framework, categorizing certainty as high, medium, low, or very low based on risk of bias, inconsistency, imprecision, indirectness, and publication bias [28]. Any disagreements between reviewers were resolved through discussion with a third researcher.

### Data extraction

Two reviewers independently extracted data from the included studies using a piloted data collection form in Excel (version 15.0; Microsoft), resolving discrepancies through discussion. The extracted information included study characteristics (first author, publication year, country, study design, study setting), participant aspects (sample size, mean age, gender, cancer type, follow-up), intervention details (aim, theoretical framework, medium, form, dose [frequency/duration], intervener), outcomes of interest (e.g., willingness to participate in cancer screening, participation rates), and specific nudging strategies.

### Data synthesis and analysis

Regarding the studies ultimately included in the analyses, heterogeneity tests and meta-analyses were conducted using RevMan 5.4 software, with a narrative synthesis for study presentation. For studies with multiple time points, the longest follow-up period was selected to assess the measurement of the outcome, with a focus on RCTs with a digital push component in the intervention group.

Heterogeneity was assessed using the* I*^2^ statistic, with *p*-values < 0.05 indicating statistical significance. *I*^2^ values of 25%, 50%, and 75% indicated low, moderate, and high heterogeneity, respectively. Significant heterogeneity (*p* < 0.05 or *I*^2^ > 50%) led to the use of a random-effects model; otherwise, a fixed-effects model was used [29]. For *I*^2^ > 50%, indicating high heterogeneity, subgroup analyses were stratified by intervention characteristics, such as cancer type, intervention medium (e.g., web vs. DVD), and delivery condition (e.g., single vs. mixed). A sensitivity analysis excluded articles with a high risk of bias. The intervention effect was calculated using the combined odds ratio (OR) and 95% confidence intervals (95% CI), which are represented as forest plots.

## Results

### Study selection

**Fig. 1:**
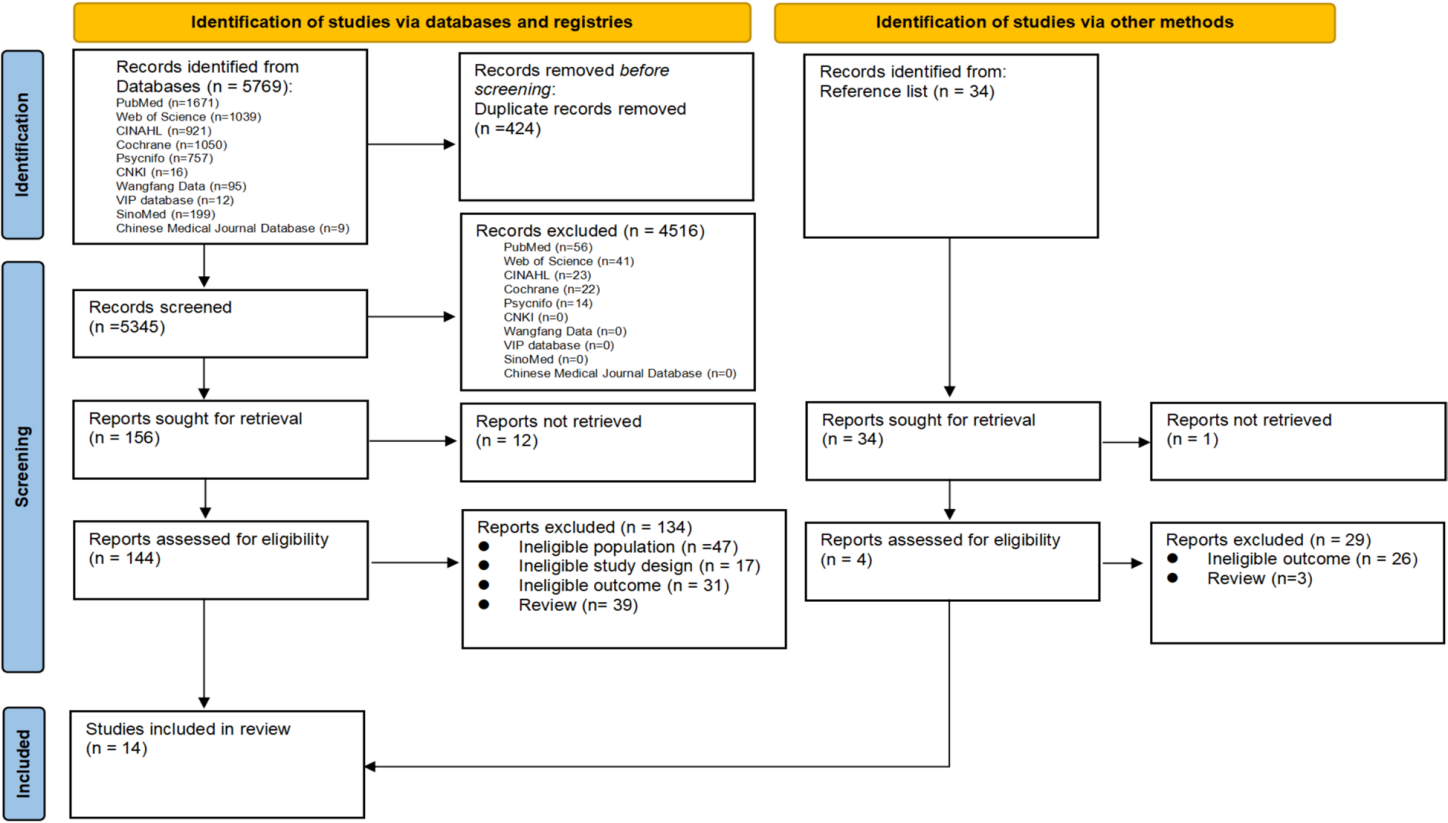
PRISMA Flow Chart of the Study Selection Process for Meta-Analysis. Abbreviations: CINAHL, Cumulative Index to Nursing and Allied Health Literature; CNKI, Chinese National Knowledge Infrastructure; VIP database, China Science and Technology Journal Database

Figure 1, presented as a PRISMA 2009 flowchart, illustrates the systematic search process used to select studies and provides details on the reasons for exclusion. A total of 5,769 records were identified from databases and registries, with 34 additional records identified through manual searches of reference lists. After removing duplicates, 5,345 records remained. Subsequent screening of abstracts and titles yielded 156 results. Finally, full-text screening resulted in the inclusion of 14 studies for analysis.

### Risk of bias of studies (Additional file 2: Figs. S1 and S2)

Among the 14 RCTs included, 3 studies (21%) did not implement blinding of participants or study personnel [30, 31], and 11 studies (79%) were found to have other evident biases [20, 21, 32–40]. One study reported neither random sequence generation nor allocation concealment and hence was considered to be at high risk for bias during the randomization process [36].

### Study characteristics

This review included randomized controlled trials (RCTs) from the United States, published between 2006 and 2024, with most (79%) published in the past decade [20, 21, 30, 33–39, 41]. Three studies (21%) were conducted in medically underserved areas [30, 34, 35], such as rural counties, whereas the majority of the studies (79%) were conducted in urban areas of communities [20, 31, 32], healthcare centers [21, 33–35], or clinics [30, 38–40]. The number of participants ranged from 120 to 1,681, with all studies including more than 100 participants. One study focused on both colorectal and breast cancer screening (7%) [34], seven focused on breast cancer screening (50%) [20, 21, 31–33, 36, 41], and six focused on colorectal cancer screening (43%) [30, 34, 37–40]. The average participant age ranged from 50.6 to 58.9 years.

Among the 14 included RCTs, all employed interactive tailored digital nudge interventions. The control groups mainly received usual care (*n* = 7, 50%) [21, 30, 33–35, 41], delayed intervention (*n* = 1, 7%) [20], or no intervention (*n* = 2, 14%) [37, 40]. Non-interactive interventions were used as controls in three studies (*n* = 3, 21%) [31, 32, 39], and one study used an interactive intervention that delivered a different message than the control (*n* = 1, 7%) [38]. Additional details about the study features are summarized in Table 2.

**Table 2:** Study characteristics

No	Author, Year, Study Country	Study Design	Setting	Sample			Type of Cancer	Intervention Group	Control Group	Follow-Up	
				Sample Size	Age Mean (SD)	Gender *n* (%)				Duration	Withdraw
1	Bowen et al., 2017, the USA [20]	Two arm RCT	Urban community	Total: 1354 IG: *n* = 677; CG: *n* = 677	NR (18–74)	Female (100%)	Breast cancer	IG: Web-based nudge intervention to influence breast health choices	CG: Delayed intervention	Baseline; 12-months	Total: 148 IG: *n* = 72; CG: *n* = 76
2	Champion et al., 2006, the USA [32]	Four arm RCT	3 urban community service centers	Total: 344 IG: *n* = 138 CG1: *n* = 71; CG2: *n* = 135	50.6 (41–75)	Female (100%)	Breast cancer	IG: Interactive computer-assisted teaching program customized to individual beliefs and stages of mammography	CG: Non-interactive group (pamphlet and video) CG1: Breast cancer screening educational pamphlets and a list of local mammography facilities CG2: Culturally appropriate videos that encourage women to adhere to mammograms	Baseline; 4 weeks; 6-months	Total: 45 IG1: *n* = 13 CG1: *n* = 15; CG2: *n* = 17
3	Champion et al., 2016, the USA [33]	Three arm RCT	Health maintenance organization or insurance plan	Total: 1681 IG1: *n* = 558; IG2: *n* = 576 CG: *n* = 547	NR (51–75)	Female (100%)	Breast cancer	IG1: Mailed tailored interactive DVD nudge intervention IG2: Computer-tailored nudge telephone counseling interventions	CG: Usual care	Baseline; 4 weeks; 6-months	Total: 43 IG1: *n* = 16; IG2: *n* = 17 CG: *n* = 10
4	Champion et al., 2018, the USA [34]	Four arm RCT	2 health care systems	Total: 1196 IG1: *n* = 303; IG2: *n* = 296 IG3: *n* = 292; CG: *n* = 305	58.9 (51–74)	Female (100%)	Colorectal cancer	IG1: Tailored web-based nudge intervention IG2: Tailored telephone intervention IG3: Tailored nudge web + telephone intervention	CG: Usual care	Baseline; 2 weeks; 6-months	Total: 275 IG1: *n* = 83; IG2: *n* = 54 IG3: *n* = 69; CG: *n* = 69
5	Champion et al., 2020, the USA [35]	Four arm RCT	2 health care systems	Total: 692 IG1: *n* = 180; IG2: *n* = 168 IG3: *n* = 167; CG: *n* = 177	58.7 (51–75)	Female (100%)	Colorectal cancer, Breast cancer	IG1: Tailored web-based nudge intervention IG2: Tailored telephone counseling IG3: Tailored nudge web + telephone intervention	CG: Usual care	Baseline; 6-months	Total: 184 IG1: *n* = 55; IG2: *n* = 35 IG3: *n* = 48; CG: *n* = 46
6	Champion et al., 2022, the USA [36]	Three arm RCT	98 rural counties	Total: 406 IG1: *n* = 157; IG2: *n* = 162 CG: *n* = 83	58.2 (50–74)	Female (100%)	Breast cancer	IG1: Tailored DVD nudge intervention IG2: Tailored DVD + patient navigation nudge intervention	CG: Usual care	Baseline; months; 12-months	Total:2 IG1: *n* = 1; IG2: *n* = 1 CG: *n* = 0
7	Fernández et al., 2015, the USA [37]	Three arm RCT	24 rural communities	Total: 656 IG1: *n* = 236; IG2: *n* = 216 CG: *n* = 204	NR	Male (30.6%)	Colorectal cancer	IG1: Small media print intervention (including educational videos and flip charts and flipchart) IG2: Interactive multimedia intervention	CG: No intervention	Baseline; 6-months	Total: 238 IG1: *n* = 93; IG2: *n* = 107 CG: *n* = 38
8	Gathirua-Mwangi et al., 2016, the USA [21]	Three arm RCT	Health maintenance organization or insurance plan	Total: 244 IG1: *n* = 87; IG2: *n* = 85 CG: *n* = 72	NR (41–65)	Female (100%)	Breast cancer	IG1: Interactive DVD nudge Intervention IG2: Tailored telephone counseling intervention	CG: Usual care	Baseline; 4 weeks; 6-months	Total: 7 IG1: *n* = 4; IG2: *n* = 2 CG: *n* = 1
9	Lee et al., 2017, the USA [41]	Two arm RCT	Underserved community	Total: 131 IG: *n* = 68; CG: *n* = 63	51.6 (40–79)	Female (100%)	Breast cancer	IG: Nudge intervention based on a mobile phone app combined with a health navigation service	CG: Usual care	Baseline; 1 week; 6-months	Total: 11 IG: *n* = 8; CG: *n* = 3
10	Rawl et al., 2021, the USA [39]	Two arm RCT	11 urban primary care clinics	Total: 817 IG: *n* = 407; CG: *n* = 410	NR (51–80)	Male (48.6%)	Colorectal cancer	IG: Tailored web-based nudge intervention	CG: Non-tailored educational brochure	Baseline; 1 weeks; 6-months	Total: 124 IG1: *n* = 72; CG: *n* = 52
11	Vernon et al., 2011, the USA [40]	Three arm RCT	19 urban clinics	Total: 1224 IG1: *n* = 413; IG2: *n* = 398 CG: *n* = 413	NR (50–70)	Male (40.8%)	Colorectal cancer	IG1: Tailored web-based nudge intervention IG2: Public web site intervention	CG: No intervention	Baseline; 2 weeks; 6-months	Total: 178 IG1: *n* = 67; IG2: *n* = 57 CG: *n* = 54
12	Rawl et al., 2024, the USA [30]	Three arm RCT	Safety-net health system	Total: 371 IG1: *n* = 123; IG2: *n* = 120 CG: *n* = 128	57.8 (45–75)	Male (39.4%)	Colorectal cancer	IG1: Mailed tailored DVD nudge intervention IG2: The mailed DVD + phone-based patient navigation nudge intervention	CG: Usual care	Baseline; 2 weeks; 6-months	Total: 63 IG1: *n* = 26; IG2: *n* = 25 CG: *n* = 12
13	Greiner et al., 2014，the USA [38]	Two arm RCT	9 urban safety-net clinics	Total: 470 IG1: *n* = 234; IG2: *n* = 236	57	Male (36.4%)	Colorectal cancer	IG: Interactive multimedia nudge interventions based on ‘implementation intentions’	CG: Interactive multimedia nudge interventions for general educational conditions	Baseline; 13–26 weeks	Total: 71
14	Russell et al., 2010，the USA [31]	Two arm RCT	Urban community health center	Total: 181 IG1: *n* = 91; CG: *n* = 90	51.2 (41–75)	Female (100%)	Breast cancer	IG: Tailored computer program nudge intervention + counseling by LHA	CG: Culturally appropriate educational pamphlet	Baseline; 6-months	Total: 36 IG1: *n* = 11; CG: *n* = 25

*Abbreviations: IG* intervention group, *CG* control group, *NR* not reported, *LHA* lay health advisor

### Characteristics of the digital nudge intervention

With respect to theoretical frameworks, eight studies used combined theories [21, 31–35, 37, 39], including the Transtheoretical Model, Health Belief Model, Behavioral Theory, Theory of Planned Behavior, Extended Parallel Process Model, and Fishbein’s Integrated Model. Four studies employed a single theoretical framework, such as the Transtheoretical Model [40], Self-Regulation Model [20], and the Precaution Adoption Process Model (PAPM) [38]. Eleven studies based their intervention programs on these theories [20, 21, 31–35, 37–40], whereas three did not specify their frameworks [30, 36, 41].

Tailored or interactive nudging strategies were implemented by trained interventionists and research assistants in twelve studies (*n* = 12, 86%) [20, 21, 30–36, 38–40] and by lay health workers in two studies (*n* = 2, 14%) [31, 37]. These strategies were delivered through various media: DVD (*n* = 6, 43%) [21, 30, 32, 33, 35–37], web or computer (*n* = 10, 71%) [20, 21, 31, 32, 34, 35, 37–40], apps (*n* = 1, 7%) [41], telephone (*n* = 6, 43%) [21, 30, 33–35], and brochure (*n* = 4, 29%) [31, 32, 35, 39]. Four studies examined multi-strategy nudges, such as tailored webpages combined with telephone interventions [34, 35] and mailed DVDs combined with patient navigation [30, 36]. More than half of the studies used multiple intervention groups to explore different intervention effects (*n*= 8, 57%) [21, 30, 33–37, 40].

All of these studies reported the effectiveness of digital nudging strategies for long term interventions in cancer screening behaviors, with the most common follow-up time being approximately 6 months (*n* = 10, 71%) [21, 31–35, 37, 39–41]. One study had a follow-up of 13–26 weeks [38] and the remaining three studies had a follow-up of 12 months [20, 30, 36]. However, regarding the intervention dosage, five studies did not disclose dosage information [20, 36–39]. Among the remaining studies, eight reported partial duration data [21, 30, 31, 40]. The duration of the study intervention session differed somewhat depending on the media type; dosages using DVDs varied from 10 to 40 min [21, 30, 32, 33], telephone interventions ranged from 11.3 to 19 min [21, 33, 34], and websites or computer programs lasted 20 to 40 min [32, 40]. Reports on intervention frequency are sparse, with only two studies providing relevant data [31, 41]. One study noted monthly telephone interventions for four months [31], while another reported a mobile application sending 8 to 21 messages per day for seven days [41]. With respect to the intervention form, most studies (71%) implemented individual digital nudge interventions [20, 21, 30, 32–36, 38, 41], whereas 29% combined individual and group interventions [31, 37, 39, 40]. Additional details are provided in Table 3.

**Table 3:** Study Characteristics and the Efficacy of the Intervention

Study	Intervention Aim	Theoretical Framework	Intervention Medium	Intervention Form	Intervention Dosage (Frequency/Duration)	Intervener	Effectiveness Primary Outcomes	Secondary Outcomes
Bowen et al., 2017, the USA [20]	To help women make better breast health choices	Self-regulation model	Web	Individual	• Web intervention: NR / NR	Trained interviewers	Mammography screening uptake in past year (IG: 82% & CG:70%)(*p* < 0.05)	• Breast self-examination (BSE) once per month (62% IG & 41% CG) (*p* < 0.05) • Interest in genetic testing (decrease of 1.6 IG & 0.1 CG)(*p* < 0.05) • Quality of life (Increase of 13 IG & 0.3 CG)(*p* > 0.05)
Champion et al., 2006, the USA [32]	To facilitating Breast Screening Behaviours among Populations in Underserved Areas	Extended Parallel Process Model, Health Belief Model, Transtheoretical Model	Computer, Pamphlet, Video,	Individual + group intervention	• Complete computer program: NR / (20–40 min) • Watch the videotape: NR / (20 min)	A project staff	Adherence of mammography screening at 6 months (27% CG & 40% IG) (*p* < 0.05)	The change of mammography stage of readiness that moved to 1 or 2 stages ( 52% IG & 36.2% CG)(*p* < 0.05)
Champion et al., 2016, the USA [33]	To increase mammography screening in women who had not received a mammogram in the last 15 months	Health Belief Model, Transtheoretical Model	DVD, Telephone,	Individual	• Interactive DVD: NR / (10 min) • Telephone intervention: NR / (11.3 min)	Trained interviewer, research assistants	Adherence to mammography screening at 6 months (IG1 & IG2 & CG)(*p* > 0.05)	NR
Champion et al., 2018, the USA [34]	To increasing Screening Adherence in Women Who Fail to Follow Colorectal Cancer Guidelines	Health Belief Model, Transtheoretical Model, Likelihood Persuasion Behavioral Theory	Web, Telephone	Individual	• Web intervention: NR / NR • Telephone intervention: NR / (19 min)	Trained interventionists, research assistants	Adherence of colorectal cancer screening test at 6 months (IG1:22.7%, IG2: 52.5%, IG3: 44.4%, CG:24.6%) (*p* < 0.05)	The effect of intervention to move the participants from precontemplation to action was significant IG1 & CG (OR = 1.81, *p* < 0.054); IG2 & CG (OR = 7.94, *p* < 0.0001); IG3 & CG (OR = 6.68, *p* < 0.0001)
Champion et al., 2020, the USA [35]	To enhance breast and colon cancer screening adherence in women who were non-adherent to both screenings	Theory of Planned Behavior, Health Belief Model, Transtheoretical Model	Web, Telephone, Brochure	Individual	• Web intervention: NR / NR • Telephone intervention: NR / (19 min)	Trained interventionists	Receiving both a mammogram and a stool test at 6 months IG1& CG(OR = 5.37, *p* = 0.025); IG2 & CG(OR = 13.56, *p* = 0.0003); IG3 & CG(OR = 17.82, *p* < 0.0001)	NR
Champion et al., 2022, the USA [36]	To increased up-to-date (UTD) breast cancer screening rates for women in rural areas	NR	DVD, Telephone	Individual	NR	Trained interventionists; patient navigator	Uptake of UTD mammograms within 12 months( IG2: 54% & CG: 30%)(*p* < 0.001)	NR
Fernández et al., 2015, the USA [37]	To develop and evaluate the effectiveness of 2 lay health worker delivered CRC screening interventions among Hispanics	Behavioral theory, Fishbein’s Integrated Model	Multimedia, DVD	Individual + group intervention	• Small Media Print Intervention: NR / NR • Tailored interactive multimedia intervention (TIMI): NR / NR	Lay health worker (LHW), research assistants	There were no statistically significant differences in CRCS at 6 months among the IGs and CG (11.9% IG1 & 18.9% IG2 & 13.3% CG) (*p* > 0.05)	• Knowledge (increase of 1.21 IG1 & 1.25 IG2 & 1.31 CG) (*p* > 0.05) • Self-efficacy (increase of 2.62 IG1 & 3.78 IG2 & 2.66 CG) ((*p* > 0.05) • Subjective norms (increase of 12.36 IG1 & 9.92 IG2 & 11.49 CG) (*p* > 0.05) • Pros (increase of 2.37 IG1 & 2.27 IG2 & 2.49 CG) (*p* > 0.05) • Susceptibility (decrease of 1.58 IG1 & 1.74 IG2 & 1.69 CG) (*p* > 0.05) • Intent (increase of 0.64 IG1 & 0.4 IG2 & 0.15 CG) (*p* > 0.05)
Gathirua-Mwangi et al., 2016,the USA [21]	To increasing Mammography Compliance in African American Women	Health Belief Model, Transtheoretical Model	Web, DVD, Telephone	Individual	• Interactive DVD: NR / (10 min) • Telephone intervention: NR / (11.3 min)	Research assistants, Researchers Counselors	Mammography uptake increased at 6 months (41% IG1 & 42% IG2 & 35% CG) (*p* = 0.6491)	• The odds of a women’s adherence to screening (Contemplation vs pre-contemplation stage, OR = 8.8, *p* < 0.05)
Lee et al., 2017, the USA [41]	To utilizing mHealth to Promote Breast Cancer Screening Behaviour among Women in an Underserved Community	NR	Mobile Apps	Individual	• mHealth Intervention: 7 days / 8–21 messages/day	Researchers, health navigator	Prevalence of screening mammography at 6 months (75% IG & 30% CG) (*P* < 0.001)	• Intention to plan a mammogram within 1 month (increase of 14% IG & 0%CG) (*P* = 0.001) • Satisfaction with the intervention (Percentage of very satisfied 40% IG & 17% CG) (*P* = 0.003) • Knowledge about breast cancer and screening (increase of 6.4 IG & 4.51CG) (*p* > 0.05) • Perceived benefits (increase of 1.3 IG & 0.75 CG) (*p* > 0.05) • Self-efficacy (increase of 1.33 IG &1.55 CG) (*p* > 0.05)
Rawl et al., 2021, the USA [39]	To increasing CRC screening for African American primary care patients	Health Belief Model, Transtheoretical Model	Web; brochure	Individual + group intervention	NR	Trained interventionists	Any CRC screening test uptake at 6 months (IG: 26.3% & CG:18.4%)(*p* < 0.05)	NR
Vernon et al., 2011, the USA [40]	To increasing CRC screening through tailored interactive interventions	Transtheoretical model	Web	Individual + group intervention	• Web intervention: NR / (23 min) • Public web intervention: NR / (17 min)	project staff, research assistants	Completion of any CRC screening test by 6 months(IG1:28% & IG2:31% & CG:30%)(*p* > 0.05)	• Stage of change: IGs more likely to be in preparation at 6 months (59% IG1 & 42% CG, *p* = 0.001; 53.9% IG2 & 42% CG, *p* = 0.033) • Knowledge scores at 6 months (IG1 2.88 & IG2 2.70 & CG 2.68)(*p* < 0.05) • Self-efficacy scores at 6 months (IG1 3.64 & IG2 3.56 & CG 3.57)(*p* = 0.05)
Rawl et al., 2024, the USA [30]	To increasing CRC Screening Rates for Low-Income and Minority Patients	NR	DVD, telephone,	Individual	• Interactive DVD: NR / (20 min) • Patient navigation: NR / NR	Trained research staff, A trained registered nurse	Uptake of any CRC screening within 12 months(30.1% IG1 & 49.2% IG2; 49.2% IG2 & 21.1% CG) (*p* < 0 .001)	• Boston Bowel Preparation Scale (BBPS) scores for participants completing colonoscopy (7.13 IG1 & 6.59 IG2 & 7.05 CG)(*p* = 0.503) • Colonoscopy related surgical anxiety (2.46 IG1 & 2.23 IG2 & 2.28 CG)(*p* = 0.559) • Satisfaction with colonoscopy procedures (3.38 IG1 & 3.60 IG2 & 3.13 CG)(*p* = 0.210)
Greiner et al., 2014, the USA [38]	To test an implementation intentions (I-I) intervention for improving CRC screening rates	Precaution Adoption Process Model (PAPM)	Touchscreen computers	Individual	NR	Health center staff, research assistants	IG had higher odds of completing colorectal cancer (CRC) screening at 26 weeks (IG & CG, AOR = 1.83) (*p* < 0.05)	• Self-efficacy (IG & CG, AOR = 1.56) (*p* < 0.05)
Russell et al., 2010, the USA [31]	To improve adherence to mammography in underserved populations	Health Belief Model, Extended Parallel Process Model, Transtheoretical Model	Computer, Pamphlet	Individual + group intervention	• Tailored computer program: NR / NR • Telephone session counselling: 1 time/month for four months / NR	Lay health worker (LHW), research assistants	Mammography screening adherence at 6 months (50.6% IG & 17.8% CG)(*p* < 0 .001)	Forward movement in stage of screening (76.3%IG & 38.5% CG)(*p* < 0 .001)

### Effects of the digital nudge intervention on screening uptake/adherence

**Fig. 2:**
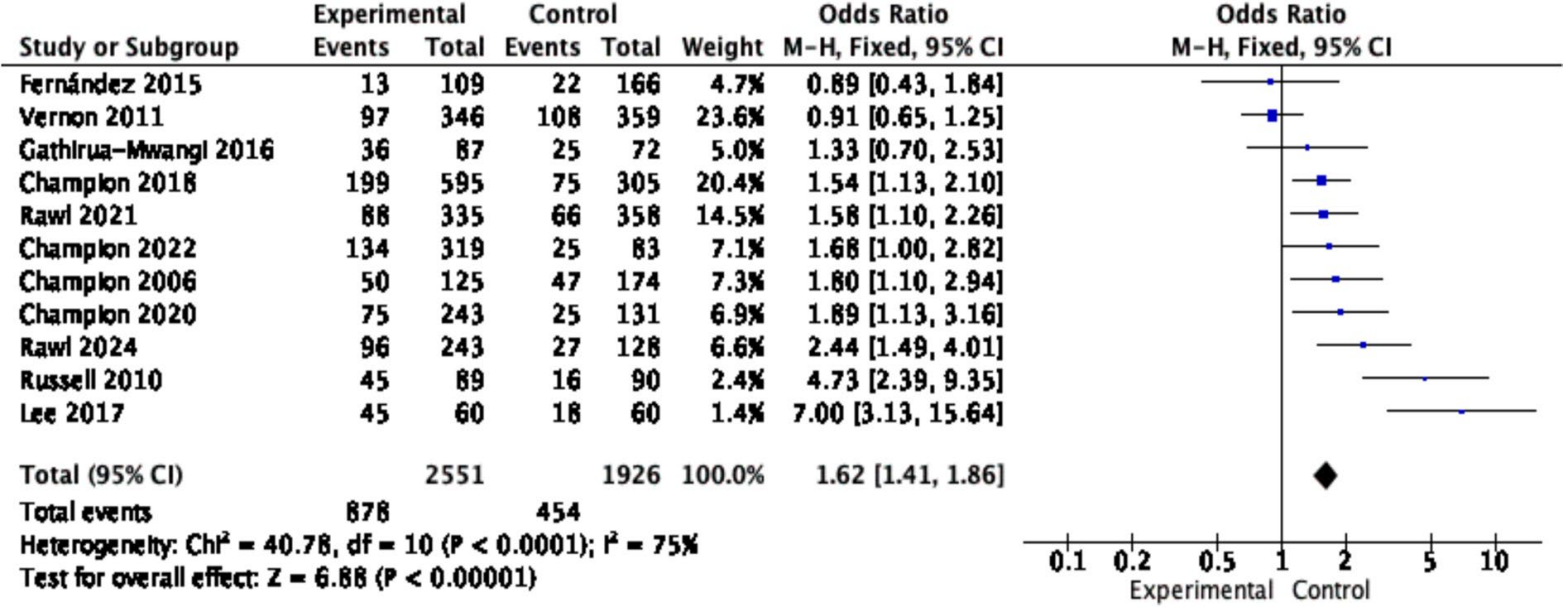
Forest plot for the rate of cancer screening in each study

There were 14 studies included in this analysis, and 3 were excluded because of missing data, resulting in a total of 11 studies included in the meta-analysis, representing 4,477 individuals at high risk of cancer. The results showed that tailored digital nudging interventions increased cancer screening uptake (OR: 1.81, 95% CI: 1.35–2.44, *p* < 0.001; Fig. 2), with moderate certainty (see Additional file 3: Table S2). However, considerable heterogeneity was observed between these studies (*I*^*2*^ = 75%). Subgroup analysis and sensitivity analysis were subsequently conducted.

#### Subgroup analysis

**Fig. 3:**
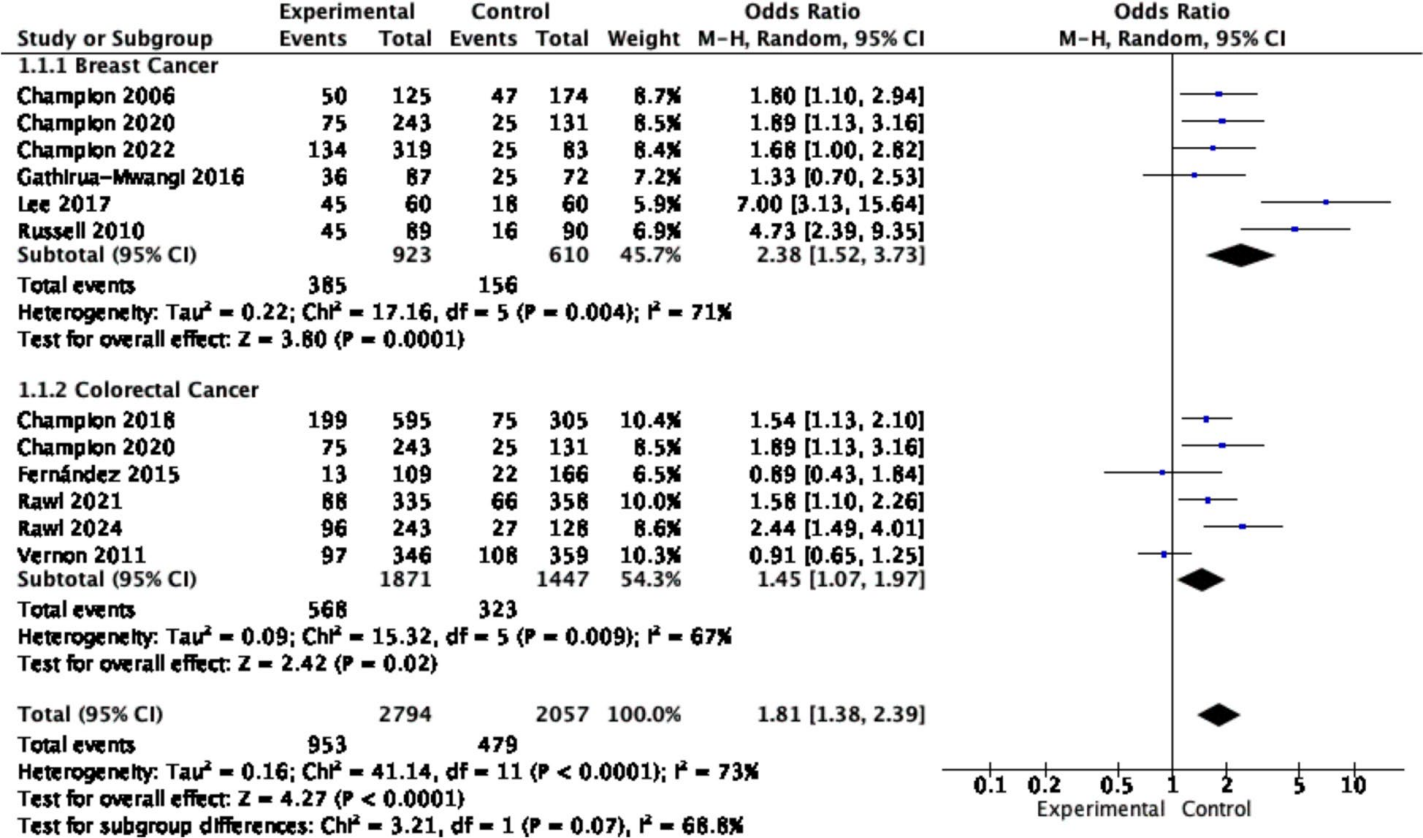
Forest plot for the rate of cancer screening by cancer type

According to subgroup analyses by cancer type, four studies focusing on breast cancer showed significant positive effects on cancer screening behaviors (OR = 2.38, 95% CI = 1.52–3.73, *p* = 0.001, *I*^*2*^ = 71%). Similarly, five studies on colorectal cancer also reported positive effects (OR = 1.45, 95% CI = 1.07–1.97, *p* = 0.02, *I*^*2*^ = 67%) (see Fig. 3).

**Fig. 4:**
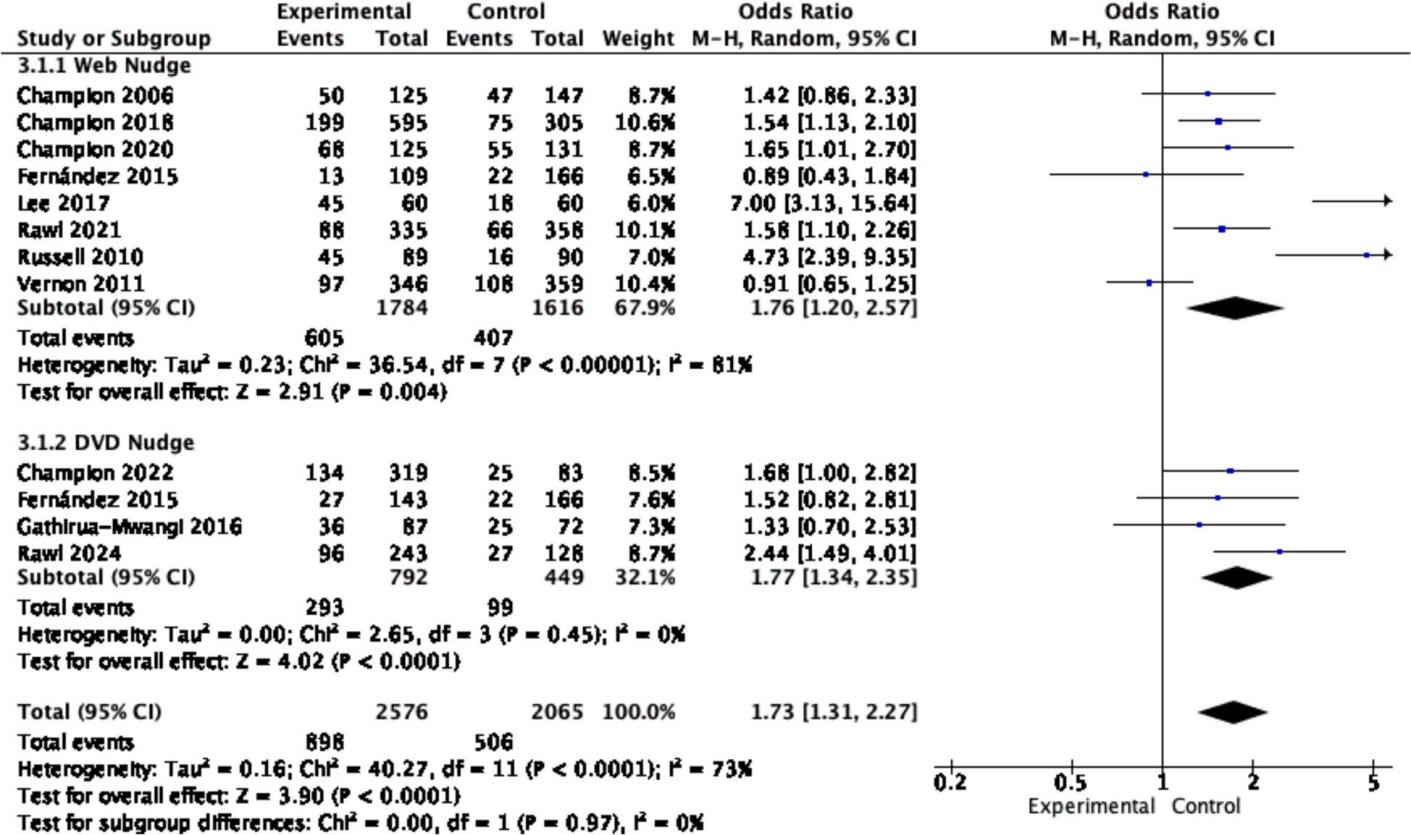
Forest plot for the rate of cancer screening by different intervention media

According to the subgroup analysis by intervention medium, digital nudging via web media had a positive effect on improving cancer screening (OR = 1.76, 95% CI = 1.20–2.57,* p* = 0.004, *I*^*2*^ = 81%), and digital nudging via DVD media had similar effects (OR = 1.77, 95% CI = 1.34–2.35, *p* < 0.001, *I*^*2*^ = 0%) (see Fig. 4).

**Fig. 5:**
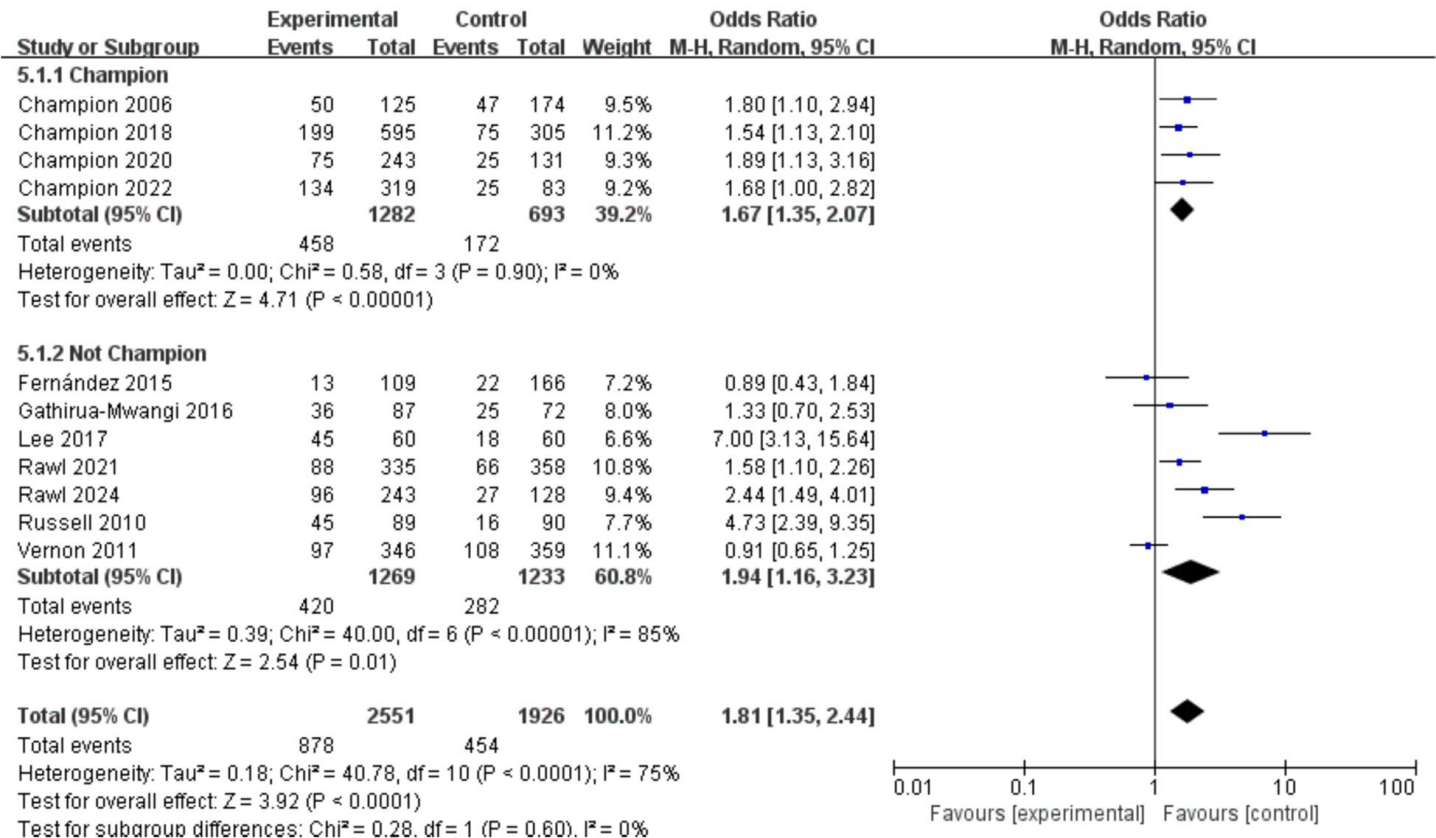
Forest plot for the rate of cancer screening by different authors

According to the subgroup analyses, studies by different authors have consistently confirmed the positive effect of digital nudge interventions in enhancing early screening behaviors among high-risk individuals (OR = 1.81, 95% CI = 1.35–2.44, *p* < 0.001, *I*^2^ = 75%). Among these, four studies conducted by Champion et al. demonstrated a significant effect with low heterogeneity (OR = 1.67, 95% CI = 1.35–2.07, *p* < 0.001, *I*^2^ = 0%). In contrast, while studies by other authors also confirmed the effectiveness of digital nudge interventions, they exhibited high heterogeneity (OR = 1.94, 95% CI = 1.16–3.23, *p* = 0.01, *I*^2^ = 85%) (see Fig. 5).

**Fig. 6:**
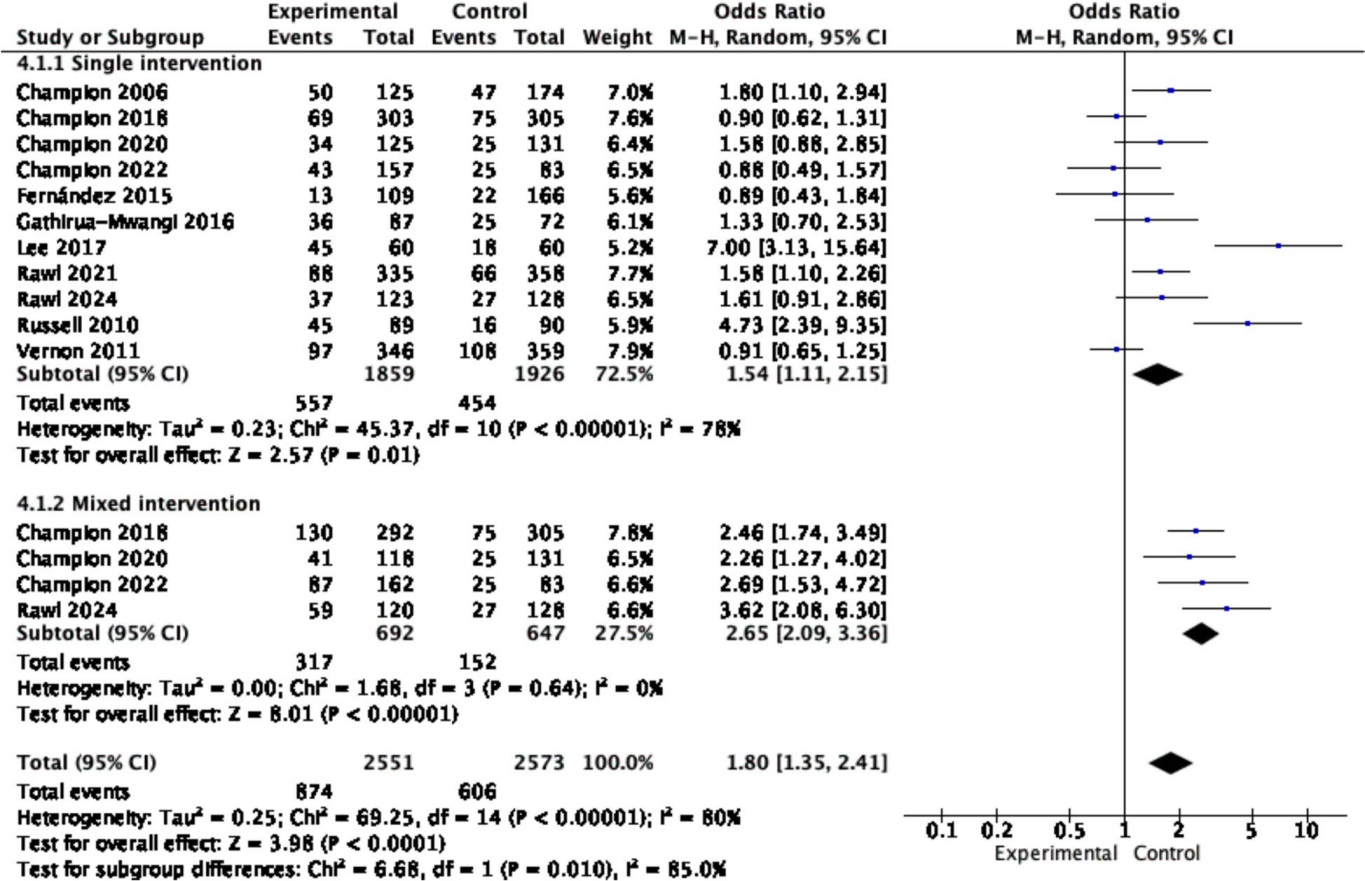
Forest plot for the rate of cancer screening by delivery condition

Subgroup analysis revealed that the single digital nudge intervention positively impacted cancer screening behaviors (OR = 1.54, 95% CI = 1.11–2.15, p = 0.01, *I*^*2*^ = 78%), and the multicomponent digital nudge intervention had an even more substantial positive effect on improving cancer screening behaviors (OR = 2.65, 95% CI = 2.09–3.36, *p* < 0.001, *I*^*2*^ = 0%) (see Fig. 6).

#### Sensitivity analysis

**Fig. 7:**
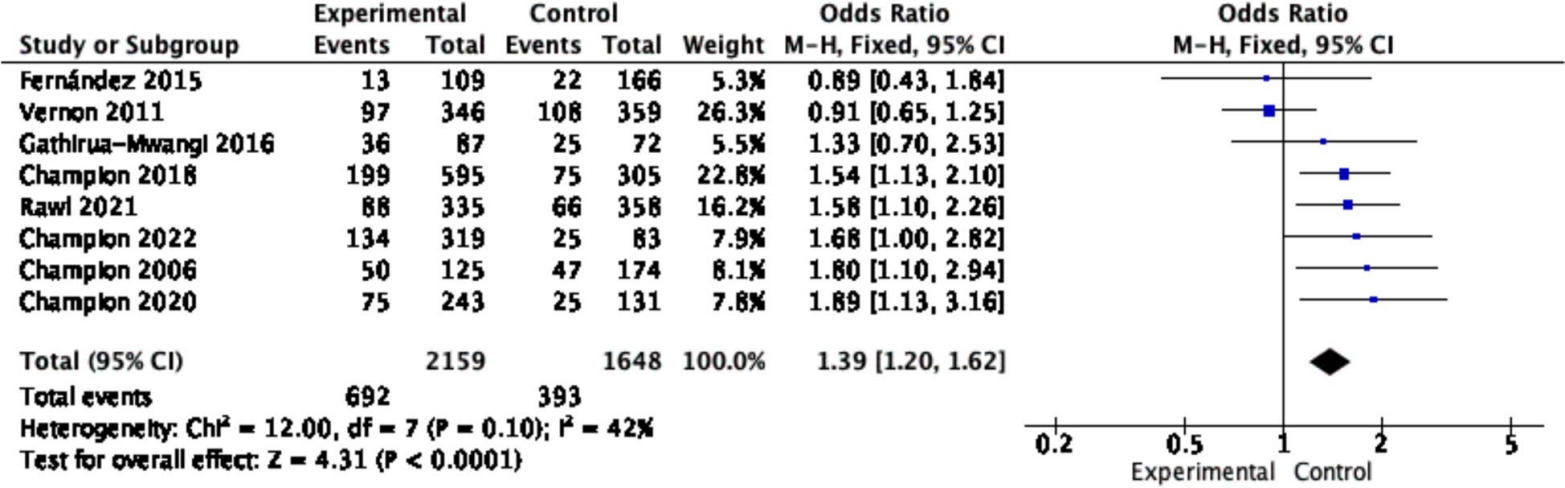
Forest plot of sensitivity estimates from included studies without a high risk of bias

Given the heterogeneity found among the studies in our subgroup analysis, sensitivity analysis was performed by excluding three studies with a high risk of bias. Following adjustment, the overall pooled effect estimate was 1.39 (95% CI: 1.20–1.62, *p* < 0.001). Importantly, we observed a significant reduction in between-study heterogeneity (*I*^*2*^ = 42%), which strengthens the confidence in the results (see Fig. 7).

### Other outcomes

#### Screening-related cognition

Outcome measures for screening-related attitudes varied across studies. Four RCTs assessed participants’ self-efficacy in attending cancer screenings [37, 38, 40, 41]. Only one study reported improved self-efficacy following an interactive nudge intervention [36], whereas the other three studies reported no significant difference between the intervention and control groups [37, 40, 41]. Some studies have also evaluated the acceptability, perceptions, and intentions related to screening [37, 41]. However, the effectiveness of tailored nudge interventions on screening intentions and attitudes remains unclear because of the limited number of studies and inconsistent results (see Table 3).

#### Knowledge

Three studies evaluated cancer patients’ knowledge of screening using various questionnaires. Only one study reported a significant improvement in screening-related knowledge among colorectal cancer patients after an interactive web-based intervention. [40] The other two studies did not outperform non-tailored studies in enhancing participant knowledge [37].

#### Stage of change

Five studies explored the current state of cancer screening [21, 31, 32, 34, 40]. Tailored nudge interventions were more effective than control interventions in advancing participants’ stages of screening readiness.

#### Health behavior change

One study reported that a tailored interactive nudge intervention effectively promoted breast self-examination in individuals at risk of developing breast cancer [20]. Another study focused on improving bowel preparation in individuals at high risk of colorectal cancer, [30] but reported no statistically significant difference between the intervention and control groups.

#### Quality of life

A study reported that a web-based nudge strategy significantly improved QOL for individuals at high risk for breast cancer [20].

### Digital MINDSPACE framework

**Fig. 8:**
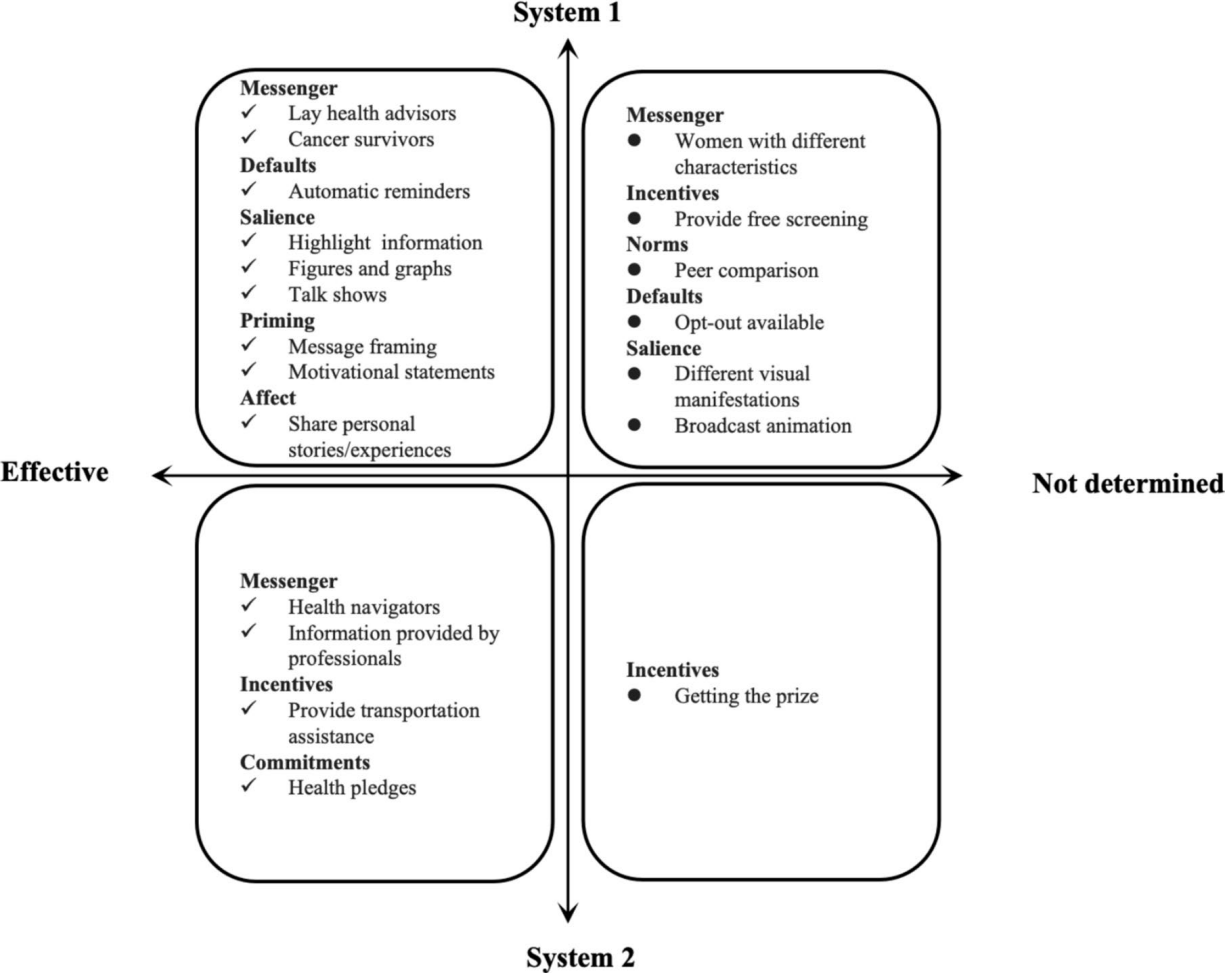
Two-dimensional nudging strategies based on MINDSPACE and heuristic-systematic models

In this paper, the cancer screening digital nudging strategies based on the MINDSPACE framework and the HSM are summarized in Table 4 and Fig. 8. Eight of the nine nudge influencers were applied throughout the interventions, with the most common nudging strategy being “default”, where a person automatically receives the nudge (*n* = 9). None of the interventions applied the ego nudge, which is based on the premise that we act and do things that enhance how we feel about ourselves.

**Table 4:** Details of the Nudge Intervention Strategies Used in Each Study

Study	MINDSPACE Framework Factor	Digital Nudge		Heuristic-Systematic Model	Was the Intervention Effective?
		Specific Strategy	Keywords		
[20]	Messenger	An expert health professional provides participants with information about the risk of breast cancer based on the questions they ask	Information provided by professional	System 2	Yes
	Defaults	A letter was sent to each participant describing the study and providing a number they could call to indicate unwillingness to participate	Opt-out available	System 1	
	Defaults	A personalized and tailored email is sent to all intervention group participants each month through a systematic reminder	Automatic reminder	System 1	
	Salience	New content has been highlighted on the homepage of the website, where participants are presented with a new breast cancer-related “Tip of the Day” each time they log on to the website	Highlighting information	System 1	
	Priming	Message framing has been altered to describe verbal messages related to breast cancer intended to increase participants’ cognitive knowledge about breast health and reduce cancer concerns. For example: • “Approximately 90% of females will never develop breast cancer.” • “Most women diagnosed with breast cancer survive beyond five years after diagnosis.” • “Heart disease causes more than eight times as many deaths as breast cancer.” • “Early detection significantly improves breast cancer treatment rates.”	Message framing	System 1	
	Salience	A visual presentation of the results of the risk prediction was given to the participants through the use of precise figures and illustrative graphs	Figures and graphs	System 1	
	Affect	Participants can access the website to view “personal stories” of different women who share their experiences with breast cancer risk	Share personal stories	System 1	
[32]	Affect	African American women shared firsthand narratives to persuade participants to reconsider health beliefs that may negatively affect adherence to mammography screening	Share personal stories	System 1	Yes
	Commitments	Participants were encouraged to take a “breast health pledge” based on the data they provided in the baseline survey. For instance, a participant who had never undergone mammography pledged to schedule one in the coming months	Health pledge	System 2	
[33]	Messenger	Four women representing different demographic profiles were chosen to deliver intervention messages	Women with different characteristics	System1	No
	Defaults	Eligible women received a letter explaining the study and were given the opportunity to call a toll-free number within two weeks if they preferred not to be contacted	Opt-out available	System 1	
	Salience	The DVD presented video and other visual representations when delivering the message	Different visual manifestations	System 1	
[34]	Incentives	Participants received a $20.00 gift certificate at each data collection time point	Getting the prize	System 2	Yes
	Defaults	Prior to contacting women, introductory letters were mailed, explaining the study and providing an opt-out opportunity through returning a postage-paid postcard or calling a toll-free number	Opt-out available	System 1	
	Defaults	The web-based program reinforces the fact that colorectal cancer can happen to anyone by automatically delivering messages to women who are not aware of the personal risk of colorectal cancer or the benefits of screening, and that screening identifies cancer early when treatment is most successful	Automatic reminder	System 1	
[35]	Defaults	Women who had not opted out by 2 weeks were called by the survey center and if they expressed interest	Opt-out available	System 1	Yes
	Salience	A web-based program featured a talk show format	Talk show (loanword)	System 1	
[36]	Messenger	A health navigator assessed participants’ knowledge and barriers related to screening to provide information about the benefits of breast cancer screening, as well as information about traveling to the clinic if necessary	Health navigator	System 2	Yes
[37]	Incentives	Participants received a $20 incentive for each survey completion	Getting the prize	System 2	No
[21]	Incentives	The cost of mammography was covered, with no co-pay or out-of-pocket funds	Provides free screening	System 1	No
	Defaults	Those who preferred not to be contacted could opt out by calling a toll-free number within two weeks of receiving the letter	Opt-out available	System 1	
	Salience	All women viewed an animation illustrating the development and spread of breast cancer throughout the body, demonstrating metastasis	Broadcasts animation	System 1	
	Messenger	The DVD began with a narrator introducing the program. Four women characters representing different demographic profiles, including an African American woman, delivered the intervention messages	Women with different characteristics	System 1	
[41]	Messenger	A health navigator was available to assist with navigating cancer screening information, addressing technical problems, and providing transportation and interpretation services	Health navigator	System 2	Yes
	Incentives	Each participant received US $20 for each face-to-face interview, plus US $20 reimbursement for text message data fees over the 6-month intervention period	Getting the prize	System 2	
	Incentives	Regardless of whether a participant answered a knowledge question correctly, she received a digital pink ribbon and collected these ribbons throughout the intervention in exchange for rewards	Getting the prize	System 2	
	Incentives	A website was created containing a list of area clinics, highlighting those offering free or discounted mammograms	Provides free screening	System 1	
	Priming	Motivational statements such as, “Call today for an appointment!” were included	Motivational statements	System 1	
	Affect	Korean American women shared their personal experiences with mammogram screening, including how they handled issues related to their cultural beliefs	Share personal stories	System 1	
	Defaults	An embedded GPS navigation system provided participants with directions and distances from their residence to their clinic of choice	Automatic reminder	System 1	
	Salience	A website was created containing a list of area clinics, highlighting those offering free or discounted mammograms	Highlighting information	System 1	
[39]	Defaults	Patients who did not call to opt-out were contacted by a recruiter who explained the study	Opt-out available	System 1	No
	Salience	The program presented an animation to illustrate the anatomy and physiology of the colon and the development of colorectal cancer from polyps	Broadcasts animation	System 1	
[40]	Incentives	All participants received $25 for attending the visit, and those completing the 6-month survey received an additional $25	Getting the prize	System 2	No
	Defaults	Patients who did not call and decline were telephoned within 2 weeks by staff and invited to enroll in the study	Opt-out available	System 1	
	Norms	The program introduced two friends discussing a mutual friend who had recently been diagnosed with colorectal cancer. The two friends were stage-matched to the study participant, with one friend being in the same stage as the participant and the other being one stage ahead	Peer comparison	System 1	
	Messenger	The DVD began with a narrator introducing the program. Four women characters representing different demographic profiles, including an African American woman, delivered the intervention messages	Women with different characteristics	System 1	
[30]	Messenger	The interactive tailored DVD’s narrative theme was a doctor’s house call to discuss ways to maintain good health, specifically through colon cancer screening	Information provided by professional	System 1	Yes
	Messenger	Two survivors of colorectal cancer provided testimonials highlighting the benefits, despite not thinking they were at risk for the disease, like many people	Cancer survivors	System 1	
	Defaults	For patients who cannot attend their screening appointment, they need to call the Endoscopy department to make a change or cancel	Opt-out available	System 1	
	Affect	Two survivors of colorectal cancer provided testimonials highlighting the benefits, despite not thinking they were at risk for the disease, like many people	Personal experience	System 1	
[38]	Incentives	Participants were reimbursed with a $20 gift card and mailed an additional $20 gift card after completion of the follow-up survey	Getting the prize	System 2	Yes
	Incentives	The study paid for a fecal immunochemical test and colonoscopies to remove the cost, a major barrier to access to screening	Provides free screening	System 2	
[31]	Messenger	Assistance to participants in capturing screening-related information through lay health advisors	Lay health advisors	System 1	Yes
	Incentives	Each participant received a $25 gift certificate after completing each survey	Getting the prize	System 2	
	Incentives	Participants received more accessible services, including referrals for low-cost or free mammograms	Provides free screening	System 1	
	Incentives	Availability of transportation assistance to participants, including free bus passes and referrals to relevant agencies	Provides transportation assistance	System 1

#### Messenger

Eight studies applied messenger nudges [20, 21, 30, 31, 33, 36, 40, 41]. These nudges included messages from expert health professionals [20, 39], cancer survivors [39], health navigators [36, 41], and lay health advisors [31], all of which effectively promoted screening behaviors. Additionally, three studies provided cancer screening information for four women with different demographic characteristics, but none of the studies reported significant results [21, 33, 40]. According to the heuristic-systematic model analyses, it was easier to quickly comprehend and receive cancer screening information through System 1 mechanisms, either through lay health navigators or women of various demographics. In contrast, System 2 mechanisms involve more deliberate processing, such as maintaining health with professionals, consulting about health-related issues, or accessing screening information with the help of a health navigator.

#### Incentives

Seven studies used incentives [21, 31, 34, 37, 38, 40, 41], predominantly pecuniary rewards, where participants received gift certificates worth $20 to $25 for completing the intervention [31, 34, 37, 38, 40, 41]. Lee et al. also offered a digital pink ribbon for answering questions that were exchangeable for a gift [41]. Additionally, four studies covered screening test costs to remove financial barriers [21, 31, 38, 41], with one providing free transportation to the screening [31]. These incentives are intended to engage System 2 processing, involving deliberate and analytical decision-making. However, whether through monetary rewards or free screening services, which might also engage intuitive System 1 thinking, the results were mixed.

#### Norms

Only one study referenced norms, using a System 1 peer comparison-based intervention [40]. It featured a program where two friends discussed a mutual friend’s colorectal cancer diagnosis, with one friend matched to the participants’ screening stage and the other one stage ahead.

#### Defaults

Nine studies on cancer screening interventions utilized default nudging, a System 1 strategy [20, 21, 30, 33–35, 39–41]. Most studies notified participants by email or text, with default participation and an opt-out option via a toll-free number. Bowen et al. used an interactive website to send monthly default newsletters to increase participation in breast cancer screening [20]. Another study used web pages to automatically deliver information to women unaware of their colorectal cancer risk or screening benefits [34]. Lee et al. [41] employed an embedded GPS navigation system to guide participants to their chosen clinics [41].

#### Salience

In six studies, diverse and appealing measures such as highlighting “free, discount” and “daily tips”; displaying figures and charts; and using videos, talk shows, and animations to demonstrate the cancer screening process, were employed to capture participants’ attention [20, 21, 33, 35, 39, 41]. These salience nudges fell under System 1 in the HSM.

#### Priming

Two studies used priming, a System 1 mechanism in which subconscious cues influence actions. Bowen et al. [20] improved participants’ perceptions of breast health by framing messages such as “early detection significantly improves breast cancer treatment rates.” [20] Lee et al. [41] used motivational statements such as “call for an appointment today!” to encourage breast cancer screening [41].

#### Affect

Four studies included affective components that engaged System 1 mechanisms and demonstrated effectiveness [20, 30, 32, 41]. These interventions sought to influence actions through emotional associations and used DVDs or videos featuring individuals who had undergone cancer screening that shared their experiences. For example, Champion et al.’s [32] featured African American women sharing personal stories that addressed health beliefs hindering mammography screening adherence [32].

#### Commitments

The effectiveness of commitments nudging has been demonstrated, even though this review included only one study that involved a commitment component. In this study, participants made a “breast health pledge” based on data from a baseline survey [32]. This commitment setting aligns more with System 2 processing.

## Discussion

### Summary of the main results

In contrast to previous reviews that focused primarily on either digital interventions or nudging strategies for individual health behaviors, our systematic review is novel in its comprehensive investigation of 14 digital nudge intervention studies. This study highlights the value of digital nudging strategies in promoting cancer screening. Our systematic review and meta-analysis demonstrated that digital interventions effectively enhance cancer screening uptake and adherence among at-risk individuals. However, the impact on other outcomes, such as cancer screening knowledge, self-efficacy, attitudes, or other health behaviors, remains inconclusive because of mixed findings and insufficient data for definitive conclusions. Notably, our review is the first to characterize digital nudge interventions using multidimensional cognitive and behavioral analytical tools, namely the MINDSPACE framework and HSM. This approach reveals potential mechanisms underlying digital nudging strategies in cancer screening.

Despite the demonstrated efficacy of digital nudge interventions highlighted in our review, critical issues and heterogeneity persist. First, the 14 studies reviewed, which focused on individuals at risk for breast and colorectal cancer in the United States, confirmed that digital nudge interventions effectively increased participation in both types of screening, with stable effect estimates, which is consistent with previous review findings [23]. However, these studies did not account for diverse cultural contexts or other cancer types, both of which are crucial factors in determining the acceptance and efficacy of screening interventions. Therefore, future studies are urgently needed to conduct more high-quality RCTs to validate the effectiveness of digital nudge interventions in promoting adherence to screening for various cancer types across different cultural contexts, thereby providing a stronger evidence base for global cancer screening and prevention efforts.

Second, existing interventions primarily target urban areas, neglecting rural and underserved regions, thereby exacerbating disparities across the health spectrum. The available evidence suggests low cancer screening participation among rural residents in high-income countries [42, 43]. For example, colorectal cancer screening rates are significantly lower in rural areas than in urban areas [42]. Therefore, there is an urgent need to consider cultural nuances and tailor digital nudges to the specific needs of diverse populations to ensure comprehensive health coverage, promote global health equity, and mitigate health disparities.

Third, evidence suggests that the form and frequency of the intervention content impacts effectiveness [44]. Consistent with previous research [45, 46], our subgroup analyses show that multicomponent intervention strategies have greater combined effect estimates than single-component strategies do. This underscores the benefits of multicomponent interventions in improving cancer screening rates. However, the number and duration of interventions vary widely across studies. In this review, five studies did not specify their intervention dosages [20, 36–39], and most provided incomplete information [21, 30–35, 40]. Only Lee et. al. (2017) detailed their intervention well, sending 8 to 21 messages per day over 7 days to participants via a mobile app [41]. Overall, information regarding the timeframe is often underreported in digital nudge interventions. Thus, future research should delineate intervention components, dose, timeframe, and duration to enhance understanding and effectiveness.

Fourth, one study in our review utilized lay health workers to administer interventions [41]. These workers are crucial in rural primary healthcare, especially during manpower shortages, as they reduce health care provider burdens and improve intervention feasibility. [47] Ensuring their competence through proper training is essential and requires the consideration of costs and resource allocation. Therefore, when lay health worker-led digital nudge interventions are implemented, evaluating local healthcare resources is vital to ensure their sustainability.

Finally, most intervention procedures that were reviewed were tailored based on theoretical frameworks to meet individual needs. Compared with non-theory-based interventions, theory-based nudge interventions provide clearer components and interrelationships. Tailoring interventions according to individual characteristics is crucial for testing their effectiveness [48]. However, the studies reviewed lacked detailed information on how these interventions were guided, such as combining different theories and selecting specific content for each concept, making reproducibility a challenge. Future research should offer more insight into how theories inform the development of tailored messages.

### Potential theoretical mechanism of digital nudge interventions

The MINDSPACE framework was applied to extract strategies involving eight core components. Our review of each intervention strategy individually revealed differing results in matching with the HSM compared with previous studies [16]. Specifically, interventions based on “norms,” “defaults,” “salience,” “priming,” and “affect” consistently aligned with the automatic, unconscious System-1 mode of thinking. Conversely, the “commitment” component relies more on logical analysis, rational judgment, and the slower System-2 mode of thinking. However, the alignment of the “messenger” and “incentive” components with either System-1 or System-2 thinking diverged from previous research, indicating a need for deeper analysis and exploration.

With respect to messenger nudging, discussions with health care professionals about maintaining health typically involve rational thinking, which aligns with previous perspectives [16]. However, when participants received cancer screening information from non-expert sources such as lay health guides or cancer survivors, their responses tended to lean toward simpler understanding and acceptance, which is indicative of System 1 thinking. Notably, the effectiveness of messengers from various demographic data in delivering screening information remains unproven, but there is evidence that individuals from lower economic backgrounds are more sensitive to messenger characteristics [49]. This underscores the importance of employing messengers from diverse backgrounds to address inequality [50].

The effectiveness of incentives in promoting screening participation varies, with mixed evidence particularly outside the United States [51, 52]. While monetary incentives, digital pink ribbons, and transport grants may somewhat stimulate participation, they often fall short in addressing the significant psychological and financial barriers associated with screening costs, especially when they are high. In such cases, individuals may lean toward System 2 reflective thinking when making screening decisions. However, the availability of free screening can offset these barriers [53], leveraging System 1 thinking to influence behavior more effectively. Russell et al. [31] support this, suggesting that upfront incentives covering screening costs may be more enticing than post-screening cash or non-cash incentives [31].

Our study revealed that the ‘default’ dimension was the most utilized contextual cueing strategy, which is consistent with the findings of Axel et al.’s previous review [54]. Default-based intervention strategies, such as opt-out options and automatic reminders, aim to maximize individual citizen benefits while influencing behavioral patterns without restricting freedom of choice, favoring System 1 thinking. These strategies have been demonstrated to enhance patient acceptance and engagement, making them particularly effective in promoting behavioral change [51].

It is worth noting that only one study in our review addressed the component of “norms”, which is not easily explained solely by rationality but is recognized to significantly influence individual behavior [55]. However, the validity of the results regarding norms was not fully verified in this study. Similarly, interventions based on “commitment” are also limited in existing research, with only one study exploring this aspect. This intervention, which encouraged patients to commit explicitly to breast health, effectively promoted cancer screening behavior among participants. Pre-commitment can be viewed as a rational and reflective action, leveraging people’s understanding of their own volitional limitations to ensure the attainment of long-term goals through commitment [56]. However, owing to the scarcity of interventions focusing on “norms” and “commitments”, a comprehensive assessment of their effectiveness remains elusive.

In our review, more than 80% of the studies incorporated multiple nudge techniques into their interventions. Research suggests that using multiple nudge interventions can effectively enhance their effectiveness [54]. For instance, Lee et al. integrated six contextual cues into a mobile phone app-based nudge intervention, resulting in a significant increase in breast cancer screening behavior among women in underserved communities [41]. Interestingly, despite the co-application of multiple nudge strategies in various studies, the consistency of their effectiveness was not uniform across studies, indicating a need for further investigations. Additionally, the use of a comprehensive set of nudging strategies complicates the evaluation of intervention effects; future studies should employ better designs and implementation strategies that lend themselves to evaluations to optimize nudging strategies.

### Limitations

The limitations of the original studies included in this review are noteworthy. First, some studies exhibited a significant risk of bias in reporting blinding of participants, potentially undermining the reliability of the scientific evidence. Second, all included studies were carried out in the United States, and the vast majority were conducted in urban areas, which may have left underserved groups out. This geographic limitation diminishes the general applicability of findings and highlights the need for studies from diverse geographic settings, including those that are underserved and face health concerns. Third, the inclusion of only breast and colorectal cancers restricted the comprehensiveness of the cancer types examined. Additionally, some of the studies had inadequacies in presenting secondary outcome indicators, or reporting intervention dosages, which impeded a comprehensive understanding of the effects of the interventions.

With respect to the limitations of this review, several points warrant consideration. First, the review scope is confined to published research findings, potentially introducing publication bias by excluding gray literature. Second, despite a comprehensive search across ten databases, non-English and non-Chinese literature was not included, potentially overlooking relevant studies. Third, the absence of relevant studies in Chinese literature databases suggests potential oversight of cultural differences and the impact of health system characteristics. Fourth, because of the methodological subjectivity of the risk of bias tool and GRADE, the evaluation may differ across individuals. Fifth, some studies were excluded from the meta-analysis process because of factors such as data unavailability or a high risk of bias within the studies themselves, thereby limiting the availability of usable data. Finally, particular attention is required in interpreting findings that are based on only two or three studies, as the reliability and generalizability of such results remain to be verified.

### Implications for practice and research

The digital nudge intervention strategy plays an essential role in enhancing cancer screening acceptance and adherence among at-risk populations, with marked effects. Of particular interest is the use of multidimensional cognitive and behavioral analysis tools, such as the MINDSPACE framework and HSM. These findings not only reveal the potential mechanisms of action of digital nudging strategies in cancer screening but also provide valuable complementary evidence and theoretical support to those of previous studies. However, little is known about the effectiveness of intervention strategies in contexts outside of the United States. Therefore, future research should be conducted in a wider geographical area, particularly in Europe and other parts of the world, to detect and validate effective interventions. Concurrently, more research is needed to determine how such interventions increase the uptake of screening. The MINDSPACE framework and HSM can help dissect the delivery mechanisms of interventions, focusing future intervention programs on the most effective components. To identify and optimize the key components of multiple facilitation strategies accurately, future research could incorporate techniques such as the theoretical domain framework (TDF) or behavior change technique (BCT) classification. These methods can systematically analyze and assess the components of facilitation strategies to provide optimized and improved strategies.

Finally, despite the effectiveness of some interventions in increasing screening recommendations, there are still many reasons why individuals do not complete screening. These underlying reasons and barriers deserve further exploration. It is critical to confront the methodological weaknesses that exist in existing research. The use of rigorous study designs, such as RCTs, is essential for establishing strong empirical evidence that digital nudge interventions improve cancer screening behaviors.

## Conclusions

The MINDSPACE framework and HSM offer a comprehensive theoretical foundation for effectively implementing digital nudging interventions in high-risk cancer populations. While these strategies have demonstrated significant benefits in promoting early screening behaviors among at-risk individuals, there is variability in their effectiveness. Further validation and exploration in future studies are necessary to ascertain their applicability beyond the United States. Additionally, conducting high-quality RCTs with longer follow-up times by using a related behavior change framework (e.g., MINDSPACE, TDF) is encouraged to generate adequate evidence for digital nudge interventions and accurately identify key components of multiple facilitation strategies.

## Supplementary Information

Additional file 1: Table S1. Table S1: Study Search Strategies.

Additional file 2: Figures S1-S2. Figure S1: Risk of Bias Summary. Figure S2: Graphical Representation of the Risk of Bias Summary.

Additional file 3: Table S2. Table S2: Grading of Recommendations Assessment, Development, and EvaluationEvidence Profile.

## Data Availability

No datasets were generated or analysed during the current study.

## Abbreviations

NLST: National Lung Screening Trial
LDCT: Low-dose computed tomography
LCS: Lung cancer screening
HSM: Heuristic-systematic model
OR: Odds ratio
RCT: Rrandomized controlled trial
PAPM: Precaution Adoption Process Model
TDF: Theoretical domain framework
BCT: Behavior change technique
PRISMA: Preferred Reporting Items for Systematic Reviews and Meta-Analyses
