# Supplementary material for: Evaluating digital nudge interventions for the promotion of cancer screening behavior: a systematic review and meta-analysis
Source: BMC Med. 2025 Apr 14;23:214. doi: 10.1186/s12916-025-04028-8 (PMC11995504; doi:10.1186/s12916-025-04028-8)
Supplement: Supplementary file 1 — Additional file 1: Table S1. Table S1: Study Search Strategies. [file 12916_2025_4028_MOESM1_ESM.docx]

**Table S1. Study Search Strategies**

| Search Item | Category | Query |
| --- | --- | --- |
| Cancer | #1 | "Neoplasms"[Mesh] |
|  | #2 | ("cancer*" OR "neoplas*" OR "malignan*" OR "tumo*" OR "carcinoma*" OR "malignant neoplasm*" OR "neoplasm, malignant" OR "neoplasms, malignant" OR "benign neoplasm*"OR "neoplasms, benign" OR "neoplasm, benign")[Title/Abstract] |
|  | #3 | #1 OR #2 |
| Screening | #4 | "Diagnosis"[Mesh] |
|  | #5 | ("Diagnose*"OR "Diagnoses and Examination*" OR "Examination and Diagnoses" OR "Examinations and Diagnoses" OR "Postmortem Diagnosis" OR "Antemortem Diagnos*" OR "Diagnosis, Antemortem" OR "Diagnoses, Antemortem")[Title/Abstract] |
|  | #6 | "Early Detection of Cancer"[Mesh] |
|  | #7 | ("Cancer Early Detection" OR "Cancer Screening" OR "Screening, Cancer" OR "Cancer Screening Test*" OR "Screening Tests, Cancer" OR "Test, Cancer Screening" OR "Tests, Cancer Screening" OR "Early Diagnosis of Cancer" OR "Cancer Early Diagnosis")[Title/Abstract] |
|  | #8 | "Mass Screening"[Mesh] |
|  | #9 | ("Mass Screenings" OR "Screening, Mass" OR "Screenings, Mass")[Title/Abstract] |
|  | #10 | ("screen*" OR "detect*" OR "scan*")[Title/Abstract] |
|  | #11 | #4 OR #5 OR #6 OR #7 OR #8 OR #9 OR #10 |
| Nudge | #12 | "Economics, Behavioral"[Mesh] |
|  | #13 | ("behavioral economic*" OR "behavioural economic*")[Title/Abstract] |
|  | #14 | "Choice behavior"[Mesh] |
|  | #15 | ("behavioral chang*" OR "behavioural chang*" OR "Behavior, Choice" OR "Behaviors, Choice" OR "Choice Behaviors" OR "Approach Behavior*" OR "Behavior, Approach" OR "Behaviors, Approach" OR "choice architecture")[Title/Abstract] |
|  | #16 | "Risk Reduction Behavior" [Mesh] |
|  | #17 | ("Behavior, Risk Reduction" OR "Behaviors, Risk Reduction" OR "Risk Reduction Behaviors" OR "Lifestyle Risk Reduction*"OR "Risk Reduction" OR "risk aversion" OR "risk avoidanc*" OR "loss aversion" OR "loss avoidanc*")[Title/Abstract] |
|  | #18 | "Patient Navigation"[MeSH] |
|  | #19 | ("navigation, patient" OR "patient navigat*" OR "navigator, patient" OR "navigators, patient" OR "patient navigator")[Title/Abstract] |
|  | #20 | ("nudg*" OR "tailor*" OR "libertarian paternalism" OR ["prospect theory](javascript:;)" OR "game theory" OR "framing effect*" OR "context effect*" OR "question-behaviour effect")[Title/Abstract] |
|  | #21 | #12 OR #13 OR #14 OR #15 OR #16 OR #17 OR #18 OR #19 OR #20 |
| Digital | #20 | "digital health"[MeSH] |
|  | #21 | ("health digital" OR "digital health technolog*" OR "health technologies digital")[Title/Abstract] |
|  | #22 | "digital technology"[MeSH] |
|  | #23 | ("digital technologies" OR "technolog* digital" OR "digital electronics")[Title/Abstract] |
|  | #24 | "Internet-Based Intervention"[MeSH] |
|  | #25 | ("internet based intervention" OR "internet-based interventions" OR "intervention, internet-based" OR "interventions, internet-based" OR "web based intervention" OR "intervention, web-based" OR "interventions, web-based" OR "web-based intervention*" OR "online intervention*" OR "intervention, online" OR "interventions, online" OR "internet intervention*" OR "Intervention, Internet" OR "Interventions, Internet")[Title/Abstract] |
|  | #26 | "Mobile Applications"[MeSH] |
|  | #27 | ("application, mobile" OR "applications, mobile" OR "mobile application" OR "app, mobile" OR "apps, mobile" OR "mobile app*" OR "Portable software app*" OR "Software App, Portable" OR "app, portable software" OR "portable software application*" OR "application, portable software" OR "software application, portable" OR "app, smartphone" OR "apps, smartphone" OR "smartphone app*" OR "portable electronic app*" OR "App, Portable Electronic" OR "electronic app, portable" OR "Application, portable electronic" OR "electronic application, portable" OR "portable electronic application*")[Title/Abstract] |
|  | #28 | ("DVD" OR "digital versatile disc" OR "digital video disc" OR "multimedia" OR "flipchart" OR "Computer-tailored" OR"eHealth" OR "mobile health" OR "health mobile" OR "mHealth")[Title/Abstract] |
|  | #29 | #20 OR #21 OR #22 OR #23 OR #24 OR #225 OR #26 OR #27 OR #28 |
| Study design | #30 | "Randomized Controlled Trials as Topic"[Mesh] |
|  | #31 | ("clinical trials randomized" OR "trials randomized clinical" OR "controlled clinical trials randomized")[Title/Abstract] |
|  | #32 | "non randomized controlled trials as topic"[MeSH] |
|  | #33 | ("controlled clinical trials non-randomized" OR "controlled clinical trials non randomized" OR "quasi-experimental stud*" OR "quasi experimental studies" OR "studies, quasi-experimental" OR "study quasi-experimental" OR "clinical trials, nonrandomized" OR "clinical trial, nonrandomized" OR "nonrandomized clinical trial*" OR "trial, nonrandomized clinical" OR "trials, nonrandomized clinical" OR "controlled clinical trials, nonrandomized" OR "clinical trials, non-randomized" OR "clinical trial, non-randomized" OR "clinical trials, non randomized" OR "non-randomized clinical trial*" OR "trial, non-randomized clinical" OR "trials, non-randomized clinical" OR "Nonrandomized controlled trials as topic")[Title/Abstract] |
|  | #34 | "cohort studies"[MeSH] |
|  | #35 | ("Cohort Study" OR "Studies, Cohort" OR "Study, Cohort" OR "Concurrent Stud*" OR "Studies, Concurrent" OR "Closed Cohort Stud*" OR "Cohort Studies, Closed" OR "Cohort Study, Closed" OR "Study, Closed Cohort" OR "Studies, Closed Cohort" OR "Birth Cohort Stud*" OR "Cohort Studies, Birth" OR "Cohort Study, Birth" OR "Studies, Birth Cohort" OR "Study, Birth Cohort" OR "Analysis, Cohort" OR "Analyses, Cohort" OR "Cohort Analys*" OR "Historical Cohort Studies" OR "Cohort Studies, Historical" OR "Cohort Study, Historical" OR "Historical Cohort Study" OR "Study, Historical Cohort" OR "Studies, Historical Cohort" OR "Incidence Stud*" OR "Studies, Incidence" OR "Study, Incidence")[Title/Abstract] |
|  | #36 | "Case-Control Studies"[Mesh] |
|  | #37 | ("Case-Control Studies" OR "Case-Control Study" OR "Study, Case-Control" OR "Case-Comparison Stud*" OR "Case Comparison Studies" OR "Studies, Case-Comparison" OR "Study, Case-Comparison" OR "Case-Compeer Studies" OR "Studies, Case-Compeer" OR "Case-Referrent Stud*" OR "Case Referrent Studies" OR "Studies, Case-Referrent" OR "Study, Case-Referrent" OR "Case-Referent Stud*" OR "Case Referent Studies" OR "Studies, Case-Referent" OR "Study, Case-Referent" OR "Case-Base Studies" OR "Case Base Studies" OR "Studies, Case-Base" OR "Case Control Stud*" OR "Studies, Case Control" OR "Study, Case Control" OR "Nested Case-Control Stud*" OR "Case-Control Studies, Nested" OR "Case-Control Study, Nested" OR "Nested Case Control Studies" OR "Studies, Nested Case-Control" OR "Study, Nested Case-Control" OR "Matched Case-Control Stud*" OR "Case-Control Studies, Matched" OR "Case-Control Study, Matched" OR "Matched Case Control Studies" OR "Studies, Matched Case-Control" OR "Study, Matched Case-Control")[Title/Abstract] |
|  | #38 | "Controlled Before-After Studies"[Mesh] |
|  | #39 | ("Before-After Studies, Controlled" OR "Before-After Study, Controlled" OR "Controlled Before After Stud*" OR "CBA Studies" OR "Controlled Before and After Studies")[Title/Abstract] |
|  | #40 | #30 OR #31 OR #33 OR #33 OR #34 OR #335 OR #36 OR #37 OR #38 OR #39 |
|  | #41 | #1 AND #2 AND (#3 OR #4) AND #5 |
