# Supplementary material for: Evaluating digital nudge interventions for the promotion of cancer screening behavior: a systematic review and meta-analysis
Source: BMC Med. 2025 Apr 14;23:214. doi: 10.1186/s12916-025-04028-8 (PMC11995504; doi:10.1186/s12916-025-04028-8)
Supplement: Supplementary file 2 — Additional file 2: Figures S1-S2. Figure S1: Risk of Bias Summary. Figure S2: Graphical Representation of the Risk of Bias Summary. [file 12916_2025_4028_MOESM2_ESM.docx]

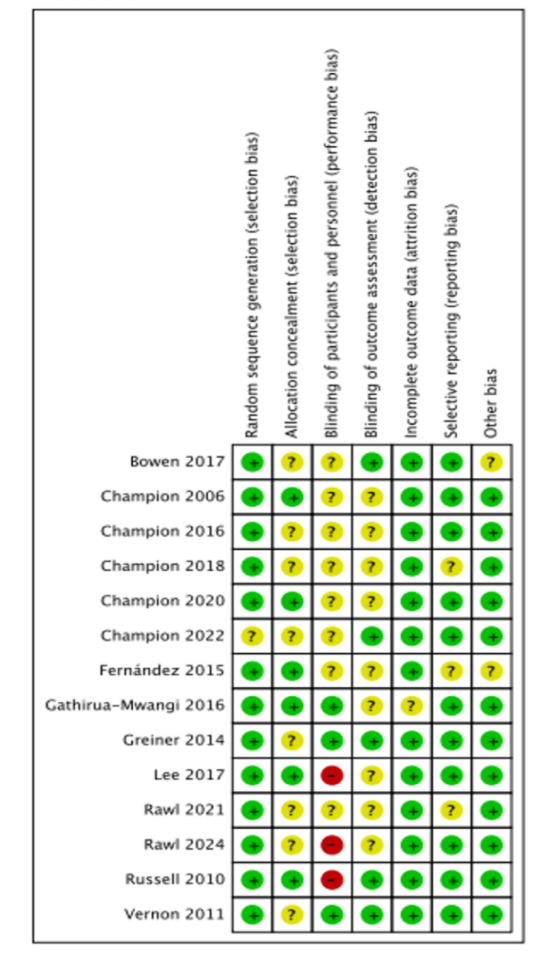


**Fig S1. Risk of Bias Summary**

Note. Green represents a low risk of bias, yellow a moderate risk, and red a high risk.


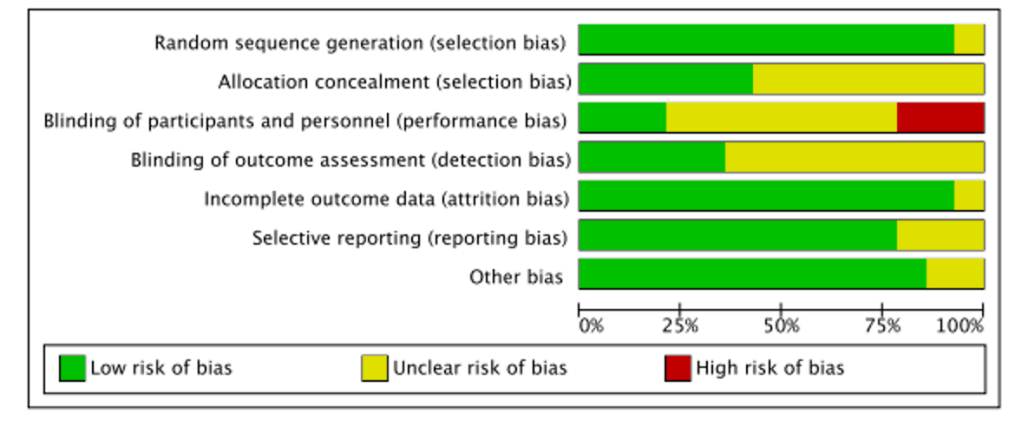


**Fig S2. Graphical Representation of the Risk of Bias Summary**
