# Supplementary material for: Evaluating digital nudge interventions for the promotion of cancer screening behavior: a systematic review and meta-analysis
Source: BMC Med. 2025 Apr 14;23:214. doi: 10.1186/s12916-025-04028-8 (PMC11995504; doi:10.1186/s12916-025-04028-8)
Supplement: Supplementary file 3 — Additional file 3: Table S2. Table S2: Grading of Recommendations Assessment, Development, and EvaluationEvidence Profile. [file 12916_2025_4028_MOESM3_ESM.docx]

**Table S2. Grading of Recommendations Assessment, Development, and Evaluation (GRADE) Evidence Profile**

| Outcomes | Risk of bias^a^ | Inconsistency^b^ | Indirectness^c^ | Imprecision^d^ | Publication bias^e^ | Certainty of the  evidence (GRADE)^f^ |
| --- | --- | --- | --- | --- | --- | --- |
| Cancer screen behaviors | Serious | Not serious | Not serious | Not serious | Not serious | ⨁⨁⨁◯ Moderate |

Note.

^a^ Risk-of-bias: certainty was downgraded if more than 50% of the weights of individual RCTs in each outcome assessed came from high-risk studies.

^b^ Inconsistency: based on the variability and heterogeneity across individual trials. Certainty was downgraded if *I^2^* > 50% and/or if the p-value of the heterogeneity test was *p*<0.05.

^c^ Indirectness: assessed qualitatively by the extent that the population, interventions, and outcome measures directly reflected the aims of the systematic review.

^d^ Imprecision: based on inspection of the pooled estimate and the 95% confidence interval (95% CIs). We decreased the grade rating by one (-1) when the analysis included fewer than 500 participants or if there were wide confidence intervals, and by two (-2) when the number of participants included in the analysis was very low or if confidence intervals were very wide.

^e^ Publication bias: assessed by a funnel plot and an extension to Egger’s regression test. Certainty downgraded when *p*<0.05.

^f^ High certainty: we are very confident that the true effect lies close to that of the estimate of the effect; Moderate certainty: we are moderately confident in the effect estimate and the true effect is likely to be close to the estimate of the effect but there is a possibility that it is substantially different; Low certainty: our confidence in the effect estimate is limited and the true effect may be substantially different from the estimate of the effect; Very low certainty: we have very little confidence in the effect estimate and the true effect is likely to be substantially different from the estimate of effect.
